# The Book World of Medicine and Science

**Published:** 1895-12-28

**Authors:** 


### THE BOOK WORLD OF MEDICINE AND SCIENCE.

Practical Ambulance Tablets. By Sidney Partridge M.D. Fourth Edition, 1895. Cloth, 12mo., pp. 60 Seven woodcuts. (London: J. and A. Churchill, 11 Burlington Street.)

It is at once evident on examining this little book that it is merely a collection of the most important principles of first aid, arranged in a tabular form to be readily carried in the pocket. It is printed in distinct type, red lettering being used to attract attention to instructions and assist ready reference. This edition differs from previous ones in that it contains the methods of carrying the sick and wounded, as taught by the St. John Ambulance Association, arranged in a succinct and easily-comprehended manner. It is a pity that no reference is made in the text to the illustrations, which are not even numbered, especially as the engraving illustrating the method of stopping bleeding from a wound of the neck, described on page 19, comes after that illustrating the method of stopping bleeding from a wound in the armpit, which is described on page 20. This is possibly an error in binding which is not present in all the copies of this issue, but all the same emphasises the advisability of the illustrations having some sort of explanation attached to them in future editions, which, from the convenient size and general utility of this work, will probably be called for before long.

Elements of Practical Medicine. By Alfred H. Carter, M.D. Seventh Edition. (London: H. K. Lewis, 1895.

This little work is so well known to all readers of medical literature, whether they be students or fully-fledged practitioners, that the appearance of a new edition is an event of some moment. The possessor of a copy of one of the earlier issues has to consider whether the progress of medicine and the possibilities for revision and improvement in Dr. Carter's "Elements" justify the outlay attendant on the purchase of a new copy. For the guidance of those who may be in doubt on this subject, and from a careful comparison of this, the last, edition with that of ten years ago, we must record our surprise and gratification at finding how closely the author has adhered to the promise he originally gave in his first preface, and how slight has been the deviation from the text. With the successive editions of many other standard works, not only in medicine, but in allied subjects also, the reader has too often to deplore a lamentable departure from the original scheme, or a sad falling off in the style. One might have reasonably anticipated a gradual development of the "Elements of Medicine" into a full-grown and austere text-book; but, *Deo gratiæ*, the book is still—to quote the author's own words in his first preface—"within the grasp of those who are not disposed or have not the leisure to read the large and complete works referred to, a class of reader which, in my opinion, meets with too little sympathy." To imply, however, that the subject matter is not fully abreast of modern and contemporary progress in medicine is to do the book a great injustice and altogether foreign to our intention. The inclusion and assimilation of so little that is new in pathology and symptomatology is by no means a flattering commentary on the value of much that appears on these subjects in the form of monographs or more ostentatious "tomes"; but that Dr. Carter has winnowed with no sparing hand and separated but little grain from the chaff is obvious to the most casual reader. In connection with what we venture to call Dr. Carter's winnowing proclivities, we find no mention under the heading of diphtheria of that fashionable and show member of this serum-therapeutic series, anti-toxin. No doubt this is a well-considered omission. In fact, the treatment generally by animal juices and organ extractives is either disregarded or but cursorily mentioned. The section on diseases of the skin is by a specialist, and not from Dr. Carter's own pen. The former has with scant success concealed his difficulties in condensing his "special" knowledge into the narrow compass of twenty-seven pages. To say that the book has not suffered damage by the process of reconstruction is to pay it a deservedly high compliment.

